# Calibration of On-Board Energy Measurement Systems Installed in Locomotives for AC Distorted Current and High Voltage Waveforms and Determination of Its Uncertainty Budget

**DOI:** 10.3390/s21237967

**Published:** 2021-11-29

**Authors:** Abderrahim Khamlichi, Fernando Garnacho, Pascual Simon, Jorge Rovira, Angel Ramirez

**Affiliations:** 1 FFII-LCOE, Eric Kandel Street 1, Getafe, 28906 Madrid, Spain; fernando.garnacho@ffii.es (F.G.); psimon@ffii.es (P.S.); jrovira@ffii.es (J.R.); angel.ramirez@ffii.es (A.R.); 2 Department of Electrical Engineering, Universidad Politécnica de Madrid, Rda. de Valencia 3, 28012 Madrid, Spain

**Keywords:** calibration uncertainty, calibration set up, fictive power source, distorted waveforms, sampling rate, energy measuring system

## Abstract

Periodic calibrations of Energy Measurement Systems (EMS) installed in locomotives must be carried out to demonstrate the required accuracy established in the EN 50463-2 standard according to European Parliament and Council Directive 2008/57/EC on the interoperability of rail systems within the Community. As a result of the work performed in the “MyRailS” EURAMET project an AC calibration facility was developed consisting of a fictive power source was developed. This fictive power source can generate distorted sinusoidal voltages up to 25 kV-50 Hz and 15 kV-16.7 Hz as well as distorted sinusoidal currents up to 500 A with harmonic content up to 5 kHz or phase-fired current waveform stated in EN50463-2 standard. These waveforms are representative of those that appear during periods of acceleration and breaking of the train. Reference measuring systems have been designed and built consisting of high voltage and high current transducers adapted to multimeters, which function as digital recorders to acquire synchronized voltage and current signals. An approved procedure has been developed and an in-depth uncertainty analysis has been performed to achieve a set of uncertainty formulas considering the influence parameters. Different influence parameters have been analyzed to evaluate uncertainty contributions for each quantity to be measured: rms voltage, rms current, active power, apparent power and non-active power of distorted voltage and current waveforms. The resulting calculated global expanded uncertainty for the developed Energy Measuring Function calibration set up has been better than 0.5% for distorted waveforms. This paper is focused on presenting the complete set of expressions and formulas developed for the different influence parameters, necessary for uncertainty budget calculation of an Energy Measuring Function calibration.

## 1. Introduction

The opening of the railway infrastructures of the different countries belonging to the European Economic Community for the free movement of trains of different companies requires the on-board measurement of electrical energy in trains to achieve the European objectives stated in [1,2]. Therefore, regular calibration and verification of on-board Energy Measuring Function (EMF) for energy billing will be a necessity for years to come. These EMF must operate within the maximum permissible error limits under actual operating conditions according to the applicable standards [3]. Strong distortions in current and voltage waveforms are caused by speed control systems (thyristors, IGBTs, etc.) of locomotives when they increase or decrease their speed by accelerating or breaking their motors. Therefore, the calculation of the uncertainty of the reference EMS used for the calibration of EMF installed in the trains for energy billing is required. This calibration must be carried out in accordance with the requirements of EN 50463-2 [3] applying voltage and current waveforms with harmonics and distortions expected in railway electrical networks [4].

The MyRailS European Project [5] has not only made it possible to develop fictitious power generation facilities in the high voltage ranges or grid frequency and harmonic content of current and voltage waveforms that are representative of the service conditions, but also it has allowed to establish a robust calibration procedure with an uncertainty of active, apparent and non-active power measurements better than 0.5%. These special calibration facilities are now available and their calibration metrological capabilities (CMC) are evaluated and presented in this paper to demonstrate traceable measurements beyond the current EURAMET recognized CMCs for any metrological institute.

The reference EMS, as part of a European project [5], was introduced and presented in [6] and a calibration setup with its capabilities was fully described in [7]. In [8], the calibration setup described in [7] was compared with another alternative, and the corresponding estimation uncertainty results were also compared. However, detailed information related to the determination of uncertainty contributions, especially those related to signal processing, were not provided. The current paper defines different installation configurations for 16.7 Hz and 50 Hz grid frequencies from the calibration configuration described in [7] and emphasizes each formula developed ad hoc for the uncertainty contribution of electrical quantities involved in the calibration procedure. In addition, a practical case of “uncertainty budget” is also developed and presented. The procedure involves transferring the voltage and current waveforms from the high voltage circuit to measurable low voltage signals, as well as using two high-precision digital multimeters as recorders that synchronously sample distorted voltage and current signals. The high voltage and high current generators used for the calibration set up generate synchronously distorted voltage and current waveforms up to 25 kV at 50 Hz or 15 kV at 16.7 Hz and up to 500 A at 50 Hz or 16.7 Hz with different frequency content up to 5 kHz to reproduce actual or normative operation conditions.

Although many authors are concerned with the measurement of active, apparent and non-active power including reactive power (see [9,10,11,12]) even of distorted alternating currents and voltages (see [13,14,15,16,17,18,19,20,21,22,23,24]), there is no literature on the evaluation of uncertainty in power measurement with harmonics or with a phase-fired current waveform as required by standard [3]. The evaluation of uncertainty due to synchronous sampling of digital multimeters has been discussed in depth in [13,14], but no study has been found in the literature on the sensitivity of power measurements to certain processing parameters, such as the integration of the signals at a time other than the full period, nor the influence of the synchronization errors between the two DMMs on the power measurement, nor the method of signal integration.

This paper also presents novel model functions for active, apparent and non-active power measurements that represent the applied measuring procedure with its influence parameters. These model functions are fundamental to establish the “uncertainty budget” through the sensitivity coefficients of each one of the influence parameters. The formulas developed in this paper for the model functions and for the determining the uncertainty together with the calculated curves that relate the influence parameters with the measurement uncertainty are the main contributions of this paper. The uncertainty analysis according to the BIPM guide [22] is presented for the developed calibration set up to determine its best Calibration Metrological Capabilities. This work is intended to contribute to future reviews of on-board EMF calibration standards to establish internal guidelines for good calibration practices that can be used by accredited calibration laboratories.

## 2. Calibration Set-Up

**Figure 1:**
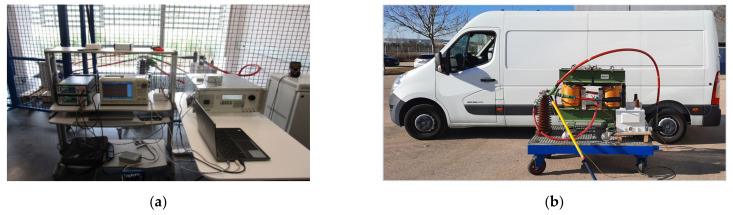
EMS calibrations according to EN 50463-2 standard: (**a**) Calibration AC facility for laboratory; (**b**) On site Calibration AC facility for on board.

**Figure 2:**
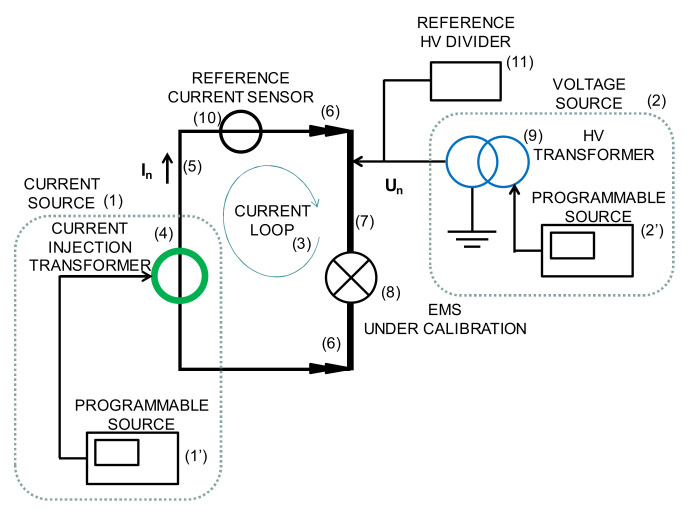
Conceptual Generation set up for 50 Hz and 16.7 Hz developed by FFII-LCOE.

**Figure 3:**
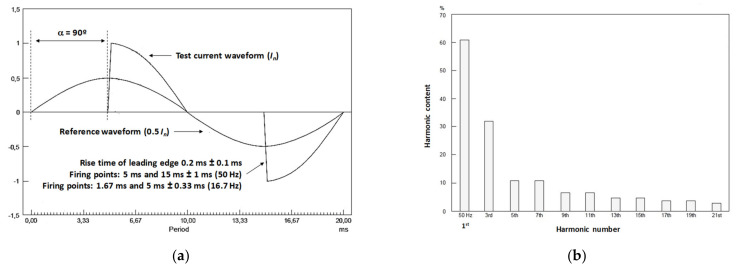
90° Phase-fired current waveform according to EN 50463: (**a**) Waveform in the time domain; (**b**) Harmonic content versus harmonic number.

**Figure 4:**
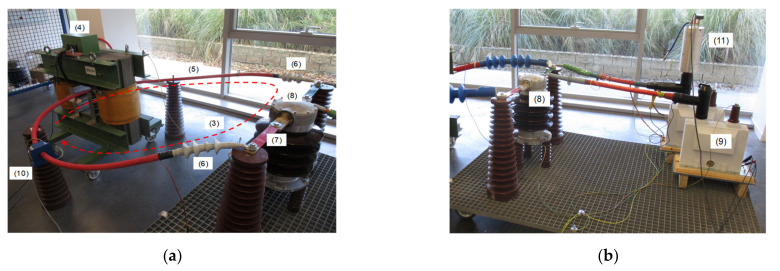
(**a**) Insulated current loop composed of a high voltage cable short-circuited by a bus bar where the EMS under calibration is installed; (**b**) High voltage source composed of two voltage transformers connected in parallel (25 kV; 50 Hz) or in series (15 kV; 16.7 Hz).

**Figure 5:**
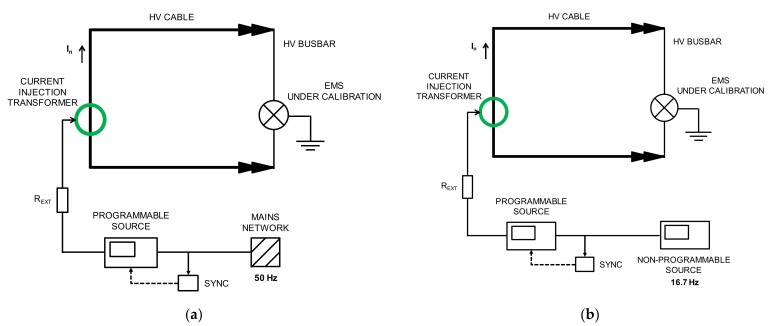
High current (up to 500 A) with harmonics injection to the current loop: (**a**) using the mains network when 50 Hz is required, (**b**) using a non-programable source when 16.7 Hz are required. Simultaneously, a sinusoidal high voltage can be applied.

**Figure 6:**
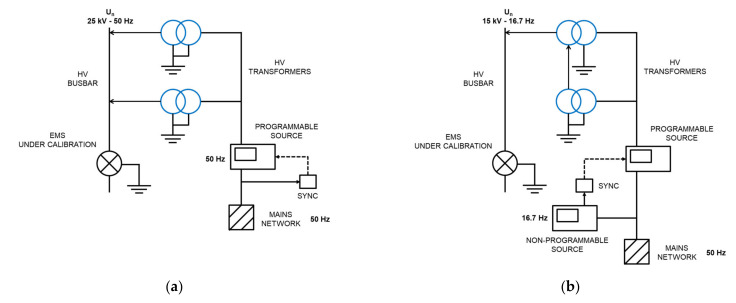
Generation of High voltage with harmonics application to the current loop, with a simultaneous injection of a sinusoidal or a phase-fired current up to 500 A: (**a**) Set up for 25 kV-50 Hz; (**b**) Set up for 15 kV-16.7 Hz.

A new calibration facility for EMS calibrations traced to national standards has been developed according to EN 50463-2 standard [3] through the European Project [5,6,7,8]. The voltage, current, and frequency values for the developed calibration set up were chosen to meet the standard requirements for laboratory calibrations or for on-site calibrations (see Figure 1). It consists of a phantom power generator (see Figure 2) composed of two independent synchronized sources: (1) a sinusoidal current source (50 Hz or 16.7 Hz) or a phase-fired current waveform up to 500 A rms (Figure 3) and (2) a high voltage source up to 25 kV, 50 Hz or 15 kV, 16.7 Hz. The current source is connected to the primary winding of a current injection transformer (4) and fed by a programmable calibration source (1′). The secondary of the current injection transformer consists of a current loop (3), composed by a high voltage cable (5) with its two cable terminations (6) short-circuited by means of a bus bar (7), where the EMS under calibration (8) is installed (see Figure 4 and Figure 5). The high voltage source (2) consists of two voltage transformers connected to the current loop, fed by a second programmable calibration source (2′). Depending on the operating conditions, the transformers are connected in parallel for 25 kV, 50 Hz to avoid excessive temperature rise in their windings, or in series for 15 kV-16.7 Hz, to avoid their magnetic saturation (see Figure 6).

This phantom generator works in the two different ways:

- Mode C “Current with harmonics”: fictive electrical power is generated by injecting a sinusoidal current (50 Hz or 16.7 Hz) or a phase-fired current waveform up to 500 A rms with harmonic content up to 5 kHz in the current loop (see Figure 5). Simultaneously, a 50 Hz or 16.7 Hz high voltage is applied.
- Mode V “Voltage with harmonics”: fictive electrical power is generated by applying a sinusoidal high voltage up to 25 kV, 50 Hz or 15 kV 16.7 Hz with harmonic content up to 5 kHz (see Figure 4) to the current loop. Simultaneously, a 50 Hz or 16.7 Hz current or a phase-fired current waveform up to 500 A is induced on the loop.

The two programmable voltage sources (1′) and (2′) are California models: type CSW5550 of 312 V, 16 A and type 5001-ix of 300 V, 16.6 A respectively. One source is connected to the low voltage winding of the high voltage transformer (9) (Arteche VEG-24 22,000/110 V, 50 Hz), while the other source is connected to primary winding of the current transformer (4) (Mimaven 160/4000 A, 50 Hz). Both current and voltage transformers were characterized in the frequency domain up to 5 kHz. This characterization is used to compensate the attenuation ratio at higher frequencies than the fundamental frequency: 50 Hz or 16.7 Hz. The frequency of the low voltage and current sources are synchronized to allow a controlled phase shift between both signals. This synchronization is performed by a device (SYNC) whose output is a trigger signal with the same frequency as the input signal. The input signal, taken as reference, can be chosen from mains network (see Figure 6a) or from another programmable or non- programmable source (see Figure 6b). The shift delay between both sources is an independent parameter that is set by a dedicated control software.

### 2.1. Reference Energy Measuring System

The reference energy measuring system performs synchronized measurements using independent voltage and current measuring systems. The current measuring system consists of a Fluxgate sensor type LEM ITN-900-S with a shunt resistor as the current transducer (10) and the high voltage measuring system consists of an improved R/C high voltage divider [25] used as a high voltage transducer (11). Two identical Keithley DMM7510 multimeters, working as recorders of 1 MS/s sampling rate, were set up to acquire both voltage and current waveforms. One multimeter works as the master, while the other is controlled by the first, working as a slave. The sampling rate and the starting time of slave DMM is the same as the master DMM and is configured by the control software.

Moreover, special software was developed to measure both voltage and current signals and to calculate the active, apparent, non-active power for sinusoidal waveforms and distorted waveforms (including harmonics and 90° phase-fired current waveform).

**Figure 7:**
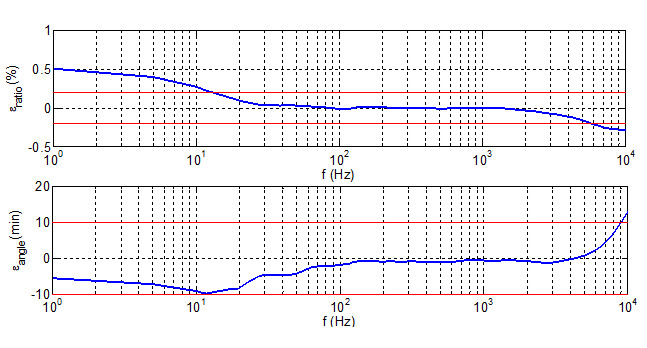
Frequency response of the Scale Factor relative error for the improved high voltage divider developed by FFII-LCOE.

**Figure 8:**
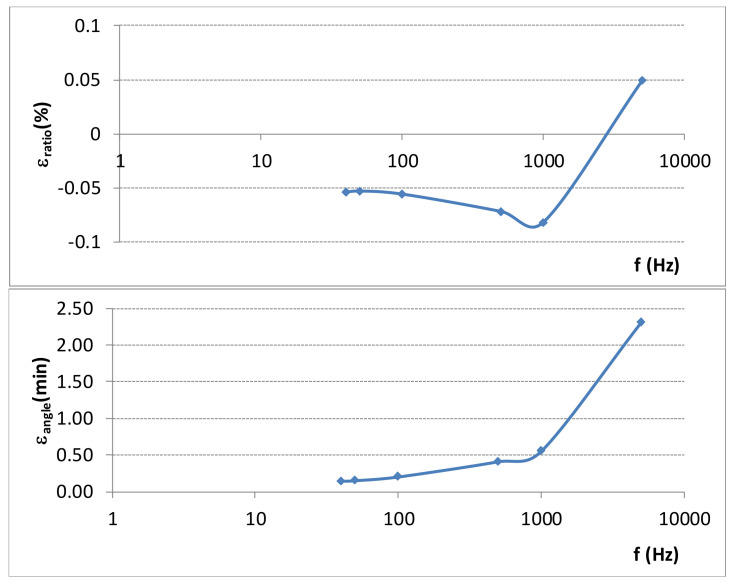
Frequency response of the Scale Factor relative error for the Fluxgate current transducer: LEM ITN-900-S.

Table 1 shows the components of both voltage and current reference measuring systems used for the calibration set up. The frequency responses of the Scale Factor relative error for the high voltage divider developed by FFII-LCOE and for the Fluxgate current transducer, are shown in Figure 7 and Figure 8, respectively.

### 2.2. Characterization and Implementation

The frequency response of current and high voltage transformers is the ratio between the output of the transformer and the input signal supplied by its California source. These transformers used in the calibration set up causes a significant attenuation and phase displacement on the signal supplied by California sources. A frequency characterization of these current and voltage transformers makes it possible to compensate the attenuation and the phase shift influence. The transformer attenuation of each harmonic component was determined during these characterizations. With this purpose, the sinusoidal signal generated by each California source was varied between the main frequency (50 Hz or 16.7 Hz) and 5 kHz by steps of the main frequency value to determine both frequency responses. The limitation for the harmonic amplitude of the voltage transformer is 5% of the main component up to 50th harmonic for 50 Hz and up to 2% for 10th harmonic, while the harmonic limitation for current transformer is not less 10% up to 50th harmonic for both 50 Hz and 16.7 Hz and more than 10% up to 100th harmonic for 16.7 Hz. These data ensure the allowable tolerance on the rise time of leading edge 0.2 ms ± 0.1 ms of the phase-fire current waveform stated in the standard EN 50,463 because not more than 4% amplitude is required up to 21st harmonic, (see Figure 3). Therefore, different correction factors were applied for each harmonic component, resulting in higher values as a function of the harmonic frequency. To achieve the required phase-fired current signal in the current transformer secondary winding, the California source generated a distorted signal considering the characterization results. More detailed information on attenuation of the injector transformers and the form of compensation is provided in [8].

The required harmonic components are measured by the current and voltage measuring systems and regulated by means of California sources considering the deviation from the target harmonic amplitudes and phase shifts. The frequency response of the relative error of the Scale factor of the High Voltage divider and of the Fluxgate current transducer are shown in Figure 7 and Figure 8 respectively.

## 3. Uncertainty Analysis

An important aim of this calibration is to verify the error of the on-board trains energy measurement systems. The calibration uncertainty should provide lower figures than the maximum permissible errors for an accepted device. The acceptable limits for the energy measurement systems, EMS, of on-board trains limits are given in [3]. Therefore, next sections are addressed to determine the calibration uncertainty of EMS calibration set up according to [8]. The definitions for electric power quantities under non-sinusoidal or unbalanced conditions are used according to [23] and studied in [24]. Appropriate signal processing treatment of the acquired AC voltage and current signals is followed according to [15,16,17,18,19,20] by synchronous digital synthesis and sampling.

### 3.1. Model Functions

#### 3.1.1. Model Functions for Rms Voltage and Current, Active, Apparent and Non-Active Power Obtained by Digital Sampling at the Low Voltage Side

A low voltage signal *v*(*t*) is sampled by means of a digital multimeter (DMM) configured for direct voltage measurements (DCV). As the voltage is sampled *n* times per period, the trapezoidal rule can be used to calculate the root mean square of the voltage *V_rms_(t_j_)* at the low voltage side for each specific period, starting at the instant *t_j_*:

(1) $$V_{rms}(t_{j})=\sqrt{\frac{1}{T}\int_{t_{j}}^{t_{j}+T}v{\left(t\right)}^{2}\cdot \;d\tau }\approx \sqrt{\frac{1}{n}\cdot \left[\sum_{k=1}^{n-1}v_{k}^{2}+\frac{v_{n}^{2}+v_{0}^{2}}{2}\right]}$$

$$\begin{array}{l} t_{j}=t_{0},t_{0}+\;T,t_{0}+2\cdot T,\;\ldots ,t_{0}+(N_{c}-1)\cdot T\;\;\; \end{array}$$

where:

*T = 1/f*: period of the power system.
*f*: fundamental frequency of the power system.
*t_0_*: time origin of the starting measuring times.
*v_0_*: acquired voltage in the first sampling interval of the period under consideration.
*v_k_*: acquired voltage in the *k^th^* sampling interval of the period under consideration.
*v_n_*: acquired voltage in the last sampling interval of the period under consideration.
*n*: Number of sample intervals per period. $n=T/h_{s}$
*h_s_ = 1/f_s_*: Sampling interval.
*f_s_*: Sampling rate.
*N_c_*: Number of periods included in the measuring time interval used to determine the energy.

Considering that the current *i(t)* measured at the low voltage side causes a voltage drop *ν’(t)* through a shunt resistance *R_s_* a similar approach can be applied to calculate the root mean square of the current *I_rms_(t_j_)*:

(2) $$I_{rms}(t_{j})=\sqrt{\frac{1}{T}\int_{t_{j}}^{t_{j}+T}i{\left(t\right)}^{2}\cdot \;d\tau }\approx \sqrt{\frac{1}{n}\cdot \left[\sum_{k=1}^{n-1}i_{k}^{2}+\frac{i_{n}^{2}+i_{0}^{2}}{2}\right]}=\frac{1}{R_{s}}\sqrt{\frac{1}{n}\cdot \left[\sum_{k=1}^{n-1}v\prime_{k}^{2}+\frac{v\prime_{n}^{2}+v\prime_{0}^{2}}{2}\right]}$$

where:

*i_k_*: acquired current in the *k^th^* sampling interval of the period under consideration.
*i_0_*: acquired current in the first sampling interval of the period under consideration.
*i_n_*: acquired current in the last sampling interval of the period under consideration.

The active power measured at the low voltage side, $P_{lv}(t_{j})$, is calculated by the following formula:

(3) $$P_{lv}(t_{j})=\frac{1}{T}\int_{t_{j}}^{t_{j}+T}v(t)\cdot i(t)\cdot dt\approx \frac{1}{n}\cdot \left[\sum_{k=1}^{n-1}v_{k}\cdot i_{k}+\frac{v_{n}\cdot i_{n}+v_{0}\cdot i_{0}}{2}\right]$$

The apparent power measured at the low voltage side, $S_{lv}(t_{j})$, is given by the formula:

(4) $$S_{lv}(t_{j})=V_{rms}(t_{j})\cdot I_{rms}(t_{j})$$

and the non-active power, $N_{lv}(t_{j})$, measured at the low voltage side by the following expression:

(5) $$N_{lv}(t_{j})=\sqrt{S_{lv}{}^{2}(t_{j})-P_{lv}{}^{2}(t_{j})}$$

#### 3.1.2. Model Functions of Voltage and Current Sampled Values at the High Voltage Side

The model function corresponding to the high voltage sample acquired at the *k^th^* sampling interval, $V_{k}$, depends on the high voltage divider and DMM.

(6) $$V_{k}=v_{k}\cdot \left(1+\delta_{v1}+\delta_{v2}\cdot \frac{V_{FS}}{\left|v_{k}\right|}\right)\cdot SF_{VD}\cdot \left(1+\underset{j}{\sum }\delta_{j,VD}\right)\cdot \left(1+\underset{j}{\sum }c_{j}\cdot \delta_{j,t}\right)$$

where:

$v_{k}$: sampled voltage value acquired at the low voltage side.
$\delta^{}{}_{v1}$: constant term of error of the DMM, for DCV measurements, typically a percentage of the voltage reading.
$\delta^{}{}_{v2}$: additional term of error of the DMM, affected by the ratio between the full-scale voltage of the DMM, $V_{FS}$, and the absolute value of the sampled voltage $\left|v_{k}\right|$.
$SF_{VD}$: calibrated scale factor of the high voltage divider.
$\delta_{j,VD}$: corrections of the voltage divider scale factor, such us drift ($\delta_{1,VD}$), temperature coefficient ($\delta_{2,VD})$, short term stability ($\delta_{3,VD}$) and non-linearity with voltage ($\delta_{4,VD}$).
$c_{j}$: sensitivity coefficient of the voltage phase displacement.
$\delta_{j,t}$: corrections of the sampled voltage value due to phase displacements. The phase shift of the DMM (negligible), the high voltage divider phase calibration ($\delta_{1,t}$), the high voltage divider phase drift (negligible), the high voltage divider residual phase correction $(\delta_{2,t}$).

The model function corresponding to the high current measurement acquired at the *k^th^* sampling interval depends on the current transducer (Fluxgate sensor), shunt and DMM.

(7) $$I_{k}=v_{k}^{´}\cdot \left(1+{\delta^{\prime }}_{v1}+{\delta^{\prime }}_{v2}\cdot \frac{{U^{\prime }}_{FS}}{\left|v_{k}^{´}\right|}\right)\cdot SF_{CT}\cdot \left(1+\underset{j}{\sum }\delta_{j,CT}\right)\cdot \frac{1}{R_{s}}\cdot \left(1+\underset{j}{\sum }\delta_{j,R_{s}}\right)\cdot \left(1+\underset{j}{\sum }c_{j}\cdot \delta^{\prime }{}_{j,t}\right)$$

where:

$v_{k}^{´}$: sampled voltage value measured at the shunt.
${\delta^{\prime }}_{v1}$: constant term of error of the DMM connected to the shunt, for DCV measurements.
${\delta^{\prime }}_{v2}$: additional term of error of the DMM connected to the shunt, affected by the ratio between the full-scale voltage of the DMM, ${V^{\prime }}_{FS}$, and the absolute value of the sampled voltage $\left|v_{k}^{´}\right|$.
$SF_{CT}$: calibrated scale factor of the current transducer.
$\delta_{j,CT}$: corrections of the current transducer scale factor, such us drift ($\delta_{1,CT}$), temperature coefficient ($\delta_{2,CT}$) and non-linearity with current $(\delta_{3,CT}$) (see note below).
$R_{s}$: calibrated value of the shunt resistor.
$\delta_{j,R_{s}}$: corrections of the calibrated shunt value, such us: drift ($\delta_{1,R_{s}}$), temperature coefficient ($\delta_{2,R_{s}}$), and variation versus frequency ($\delta_{3,R_{s}}$).
$c_{j}$: sensitivity coefficient of the voltage phase displacement in the current circuit.
${\delta^{\prime }}_{j,t}$: corrections of the sampled voltage value due to phase displacements. The phase shift of the DMM (negligible), the current sensor phase calibration (${\delta^{\prime }}_{3,t}$), the current sensor phase drift (${\delta^{\prime }}_{4,t}$), the current sensor residual phase correction (${\delta^{\prime }}_{5,t}$) and the shunt phase error (${\delta^{\prime }}_{6,t}$).

Note: The effect of leakage flux from the current transformer on the scale factor of the current sensor is negligible (lower than 0.0025%).

Amplitude corrections in the scale factor for the harmonic components (Figure 7 and Figure 8) are not considered in Formulas (6) and (7) because amplitude of harmonic components bigger than 5th is not more than 10% of main component and its scale factor correction due to frequency dependence is less than 0.1% (see Figure 7 and Figure 8). Consequently, these uncertainty contributions can be neglected. For 3rd harmonic component the scale factor is very close to the rated scale factor for the main frequency and no correction is needed.

#### 3.1.3. Model Functions for the Quantities at the High Voltage Side

The function model for the square of the rms voltage value calculated at the high voltage side is defined by the following formula:

(8) $$V_{RMS}^{}(t_{j})=\sqrt{\frac{1}{n}\cdot \left[\sum_{k=1}^{n-1}V_{k}^{2}+\frac{V_{n}^{2}+V_{0}^{2}}{2}\right]}\cdot \left[1+\sum_{l}Sp_{l}^{}\right]$$

where:

$\sqrt{\frac{1}{n}\cdot \left[\sum_{k=1}^{n-1}V_{k}^{2}+\frac{V_{n}^{2}+V_{0}^{2}}{2}\right]}$ is the rms voltage value calculated by trapezoidal rule from a finite number of samples *V_k_ (k =* 1: *n)* taken from the recorded voltage signal.

*Sp_l_*: are the corrections of the rms voltage value due to signal processing restrictions, such as finite sampling rate ($Sp_{1}$) and integration time different of the period ($Sp_{2}$). The uncertainty contribution of these terms is described in Sections Uncertainty Contribution Due to Trapezoidal Integration Rule ($Sp_{1}$) and Uncertainty Contribution Due to an Integration Time Different to the Complete Period ($Sp_{2}$), respectively.

Similarly, the function model for the current rms value calculated at the high voltage side will be:

(9) $$I_{RMS}^{}(t_{j})=\sqrt{\frac{1}{n}\cdot \left[\sum_{k=1}^{n-1}I_{k}^{2}+\frac{I_{n}^{2}+I_{0}^{2}}{2}\right]}\cdot \left[1+\sum_{l}Sp_{l}^{\prime }\right]$$

where:

$\sqrt{\frac{1}{n}\cdot \left[\sum_{k=1}^{n-1}I_{k}^{2}+\frac{I_{n}^{2}+I_{0}^{2}}{2}\right]}$ is the current rms value calculated by trapezoidal rule from a finite number of samples *I_k_ (k = 1:n)* taken from the recorded voltage signal.

$Sp\prime_{l}$: are the corrections of the rms current value due to signal processing restrictions, such as finite sampling rate ($Sp\prime_{1}$) and integration time different of the period ($Sp\prime_{2}$). The uncertainty contribution of these terms is described in Sections Uncertainty Contribution Due to Trapezoidal Integration Rule ($Sp_{1}$) and Uncertainty Contribution Due to an Integration Time Different to the Complete Period ($Sp_{2}$), respectively.

The apparent power at the high voltage side, *S_HV_*(*t_j_*), is directly determined from the rms voltage and current values determined by (8) and (9):

(10) $$S_{HV}(t_{j})=V_{RMS}(t_{j})\cdot I_{RMS}(t_{j})$$

The function model for the active power measured at the high voltage side by acquisition of voltage and current samples is given by the following expression:

(11) $$P_{HV}\left(t_{j}\right)=\frac{1}{n}\cdot \left[\overset{n-1}{\underset{k=1}{\sum }}V_{k}\cdot I_{k}+\frac{V_{n}\cdot I_{n}+V_{0}\cdot I_{0}}{2}\right]\cdot \left[1+\sum_{l}Sp_{l}+\sum_{l}Sp_{l}^{\prime }+Sp_{3}\right]$$

where:

Sp_3_: correction of the active power value due to synchronization error between both DMM.

And the non-active power, *N_HV_*(*t_j_*), will be given by:

(12) $$N_{HV}(t_{j})=\sqrt{S_{HV}{}^{2}(t_{j})-P_{HV}{}^{2}(t_{j})}$$

### 3.2. Uncertainty Analysis

#### 3.2.1. Uncertainty of the Calculated Rms Voltage and Current Values at the High Voltage Side

Considering Formula (8) as the function model of rms voltage value, considering the auxiliary function *y(t_j_)* and assuming as the best estimations of $Sp_{1}$ and $Sp_{2}$ equal to zero:

(13) $$y(t_{j})=\frac{1}{n}\cdot \left[\sum_{k=1}^{n-1}V_{k}^{2}+\frac{V_{n}^{2}+V_{0}^{2}}{2}\right]\approx V_{RMS}^{2}(t_{j})$$

The $V_{RMS}(t_{j})$ uncertainty can be expressed by:

(14) $$u[V_{RMS}(t_{j})]=\sqrt{{\left[\frac{\partial V_{RMS}(t_{j})}{\partial y(t_{j})}\right]}^{2}\cdot u^{2}[y(t_{j})]+V_{{}_{RMS}}^{2}(t_{j})\cdot \sum_{l}u^{2}(Sp_{l})}$$

where:

(15) $$\frac{\partial V_{RMS}(t_{j})}{\partial y(t_{j})}=\frac{1}{2\cdot V_{RMS}(t_{j})}$$

Taking into account the expression given in (6) as the function model of each voltage sample, $V_{k}$, measured at the high voltage side in the sampling interval *k^th^* and assuming a fully correlation between voltage measurements (correlation coefficient = 1), each calculated rms voltage uncertainty at the high voltage side, *V_RMS_(t_j_)*, can be expressed in per unit value (p.u.):

(16) $$u_{pu}[V_{RMS}(t_{j})]=\sqrt{u^{2}(\delta_{v1})+{\left[\frac{V_{ARV}^{}(t_{j})}{V_{RMS}^{}(t_{j})}\cdot \frac{V_{FS}^{}\cdot SF_{VD}}{V_{RMS}^{}(t_{j})}\right]}^{2}\cdot u^{2}(\delta_{v2})+u^{2}(SF_{VD})+\sum_{l}u^{2}(\delta_{l,VD})+\sum_{l}c_{l}^{2}\cdot u^{2}(\delta_{l,t})+\sum_{l}u^{2}(Sp_{l})}$$

where the average rectified high voltage value is:

(17) $$V_{ARV}(t_{j})=SF_{VD}\cdot \frac{1}{n}\cdot \left(\sum_{k=1}^{n-1}\left|v_{k}\right|+\frac{\left|v_{n}\right|+\left|v_{0}\right|}{2}\right)$$

$u(\delta_{v1})$: represents the uncertainty in p.u. of the correction term *δ_v1_.*
$u(\delta_{v2})$: represents the uncertainty in p.u. of the correction term *δ_v2_.*
$u\left(SF_{VD}\right)$: represents the calibration uncertainty in p.u. of the high voltage divider scale factor,$\;SF_{VD}$.
$u(\delta_{l,VD})$: represents the uncertainty in p.u. factor *δ_j,VD_.*
$u(\delta_{l,t})$: represents the uncertainty in p.u. of the correction terms related to the phase displacements due to the digitizer and the divider.
$c_{l}^{}$: represents the sensitivity coefficient of each *l^th^* correction term.
$u(Sp_{l})$: represents the uncertainty in p.u. of the correction terms related to the signal processing restrictions related to a rms of the voltage in a period T starting at *t_j_*.

Considering a sinusoidal voltage waveform with a low harmonic content (THD < 2%):

(18) $$\frac{V_{ARV}(t_{j})}{V_{RMS}(t_{j})}=\frac{1}{FF_{v}(t_{j})}\approx 2\cdot \frac{\sqrt{2}}{\pi }\approx 0.9$$

The final formula of the uncertainty expressed in p.u. using the form factor of the voltage signal in the period *T* starting at the *t_j_* instant, *FF_v_(t_j_)*, is the following, where *FF_v_(t_j_)* ≈ 1.11.

(19) $$u_{pu}[V_{RMS}(t_{j})]=\sqrt{u^{2}(\delta_{v1})+\frac{V_{FS}^{2}\cdot SF_{{}_{VD}}^{2}}{1.11{}^{2}\cdot V_{RMS}^{2}(t_{j})}\cdot u^{2}(\delta_{v2})+u^{2}(SF_{VD})+\sum_{l}u^{2}(\delta_{l,VD})+\sum_{l}c_{l}^{2}\cdot u^{2}(\delta_{l,t})+\sum_{l}u^{2}(Sp_{l})}$$

Applying the same considerations and assumptions as the ones followed for the uncertainty of a calculated rms voltage value, the uncertainty of a calculated rms current value can be expressed by the formula:

(20) $$u_{pu}[I_{RMS}(t_{j})]=\sqrt{u^{2}(\delta_{v1}^{\prime })+\frac{I_{FS}^{2}\cdot SF_{{}_{CT}}^{2}}{FF_{i}^{2}(t_{j})\cdot I_{RMS}^{2}(t_{j})}\cdot u^{2}(\delta_{v2}^{\prime })+u^{2}(SF_{CT})+\sum_{l}u^{2}(\delta_{{}_{l,CT}}^{})+u^{2}(R_{s})+\sum_{l}u^{2}(\delta_{{}_{l,R_{s}}}^{})+\sum_{l}c_{l}^{,2}\cdot u^{2}(\delta_{l,t}^{\prime })+\sum_{l}u^{2}(Sp_{{}_{l}}^{\prime })}$$

where:

(21) $$I_{ARV}\left(t_{j}\right)=SF_{CT}\cdot I_{arv}\left(t_{j}\right);\;I_{RMS}\left(t_{j}\right)=SF_{CT}\cdot I_{rms}\left(t_{j}\right);\;\;I_{FS}=\frac{V_{FS}^{\prime }}{R_{s}}$$

$u\left(SF_{CT}\right)$: represents the calibration uncertainty in p.u. of the current transducer scale factor,$\;SF_{CT}$.
$u(\delta_{{}_{l,CT}}^{\prime })$: represents the uncertainty of the correction term related to the current transducer *δ_l,CT_.*
$u\left(R_{s}\right)$: represents the calibration uncertainty in p.u. of the current shunt,$\;R_{s}$.
$u(\delta_{{}_{l,t}}^{\prime })$: represents the uncertainty of the correction terms related to the phase displacements due to current transducer, shunt resistance, digitizer.
$c_{l}^{\prime }$: represents the sensitivity coefficient of each *l^th^* correction term.
$u(Sp_{l}^{\prime })$: represents the uncertainty in p.u. of the correction terms related to the signal processing restrictions related to a rms of the current in a period T starting at *t_j_*.

When injecting a sinusoidal current waveform with a low harmonic content (THDr < 2%) the form factor to be applied in the expression (20) will is $FF_{i}\left(t_{j}\right)\approx 1.11$.

For a phase-fired current waveform of angle *α*, as shown in Figure 3, the quotient between the rectified average current and its rms value in a period follows the expression:

(22) $$\begin{array}{l} \;\;\;\;\;\;\;\;\;\;\frac{I_{arv}^{}(t_{j})}{I_{rms}^{}(t_{j})}=\frac{\sqrt{2}}{\pi }\cdot \frac{1+\cos(\alpha )}{\sqrt{\left(1-\frac{\alpha }{\pi })+\frac{1}{2\cdot \pi }\cdot \sin(2\cdot \alpha \right)}}\\ where:\;I_{arv}^{}(t_{j})=\frac{I_{pk}}{\pi }\cdot \left[1+\cos(\alpha )\right]\;\;\;\;\;\;\;\;I_{rms}^{}(t_{j})=\frac{I_{pk}}{\sqrt{2}}\cdot \sqrt{\left(1-\frac{\alpha }{\pi })+\frac{1}{2\cdot \pi }\cdot \sin(2\cdot \alpha \right)}\;\;\;\; \end{array}$$

For example, for the case of a *α =* 90° phase-fired waveform the form factor is:

$$FF_{i}\left(t_{j}\right)=\frac{I_{rms}\left(t_{j}\right)}{I_{arv}\left(t_{j}\right)}\approx 1.56$$

#### 3.2.2. Uncertainty of the Calculated Power Quantities at the High Voltage Side

##### Active Power

Considering Formula (11) as the function model of the active power and assuming as the best estimations of $Sp_{3}$ equal to zero:

(23) $$u[P_{HV}(t_{j})]=\sqrt{\sum_{k}{\left(\frac{\partial P_{HV}(t_{j})}{\partial V_{k}}\right)}^{2}\cdot u^{2}(V_{k})+\sum_{k}{\left(\frac{\partial P_{HV}(t_{j})}{\partial I_{k}}\right)}^{2}\cdot u^{2}(I_{k})+P_{{}_{HV}}^{2}(t_{j})\cdot \sum_{l}u^{2}(Sp_{l})+P_{{}_{HV}}^{2}(t_{j})\cdot \sum_{l}u^{2}(Sp_{l}^{\prime })+P_{{}_{HV}}^{2}(t_{j})\cdot u^{2}(S_{p3})}$$

To simplify editing, this expression is divided in four uncertainty terms: the first term is related to independent samples at low voltage side of both signals: voltage and current. The second and third terms are related to the high voltage and high current scale factors, respectively, and the fourth term to the signal processing:

(24) $$u_{pu}[P_{HV}(t_{j})]=\sqrt{u_{{}_{1}}^{2}+u_{{}_{2}}^{2}+u_{{}_{3}}^{2}+u_{{}_{4}}^{2}}$$

Assuming a correlation coefficient among sampled measurements of +1 the first uncertainty term can be expressed in per unit value (p.u.) as:

(25) $$u_{1}=\sqrt{\left[u^{2}(\delta_{v1})+u^{2}(\delta_{v1}^{\prime })\right]+\frac{V_{FS}^{2}\cdot I_{arv}^{2}}{P_{lv}^{2}(t_{j})}\cdot u^{2}(\delta_{v2})+\frac{I_{FS}^{2}\cdot V_{arv}^{2}}{P_{lv}^{2}(t_{j})}\cdot u^{2}(\delta_{v2}^{\prime })}$$

This expression can be particularized for sinusoidal current with low harmonic content and for 90° phase-fired current waveform.

The active power at the low voltage side for a sinusoidal voltage or current with low harmonic content can be calculated by the formula:

(26) $$P_{lv}^{}(t_{j})\approx I_{rms\_1}^{}(t_{j})\cdot V_{rms\_1}^{}(t_{j})\cdot cos\varphi_{}^{}$$

and the *u_1_* uncertainty term can be expressed by the formula:

(27) $$u_{1}=\sqrt{\left[u^{2}(\delta_{v1})+u^{2}(\delta_{v1}^{\prime })\right]+\left[\frac{1}{FF_{{}^{i}}^{2}(t_{j})\cdot {cos}^{2}\varphi }\cdot \frac{V_{FS}^{2}}{V_{rms}^{2}(t_{j})}\cdot u^{2}(\delta_{v2})+\frac{1}{FF_{v}^{2}(t_{j})\cdot {cos}^{2}\varphi }\cdot \frac{I_{FS}^{2}\cdot }{I_{rms}^{2}(t_{j})}\cdot u^{2}(\delta_{v2}^{\prime })\right]}$$

For the case of α phase-fired current waveform and an undistorted sinusoidal voltage with angle phase shift of *ϕ* = 0° between voltage and current, the active power can be calculated by:

(28) $$P_{lv}^{}(t_{j})=\sqrt{\left(1-\frac{\alpha }{\pi })+\frac{1}{2\cdot \pi }\cdot sin(2\cdot \alpha \right)}\cdot I_{rms}^{}(t_{j})\cdot V_{rms}^{}(t_{j})$$

For *α* = 90°:

(29) $$P_{lv}^{}(t_{j})\approx 0.707\cdot I_{rms}^{}(t_{j})\cdot V_{rms}^{}(t_{j})$$

and using the form factor for voltage and current 1.11 and 1.56 respectively, the *u_1_* uncertainty term will be:

(30) $$u_{1}=\sqrt{\left[u^{2}(\delta_{v1})+u^{2}(\delta_{v1}^{\prime })\right]+\left[\frac{1}{1.56^{2}\times 0.707^{2}}\cdot \frac{V_{FS}^{2}}{V_{rms}^{2}(t_{j})}\cdot u^{2}(\delta_{v2})+\frac{1}{1.11^{2}\times 0.707^{2}}\cdot \frac{I_{FS}^{2}\cdot }{I_{rms}^{2}(t_{j})}\cdot u^{2}(\delta_{v2}^{\prime })\right]}$$

resulting:

(31) $$u_{1}=\sqrt{\left[u^{2}(\delta_{v1})+u^{2}(\delta_{v1}^{\prime })\right]+\left[0.82\cdot \frac{V_{FS}^{2}}{V_{rms}^{2}(t_{j})}\cdot u^{2}(\delta_{v2})+1.62\cdot \frac{I_{FS}^{2}\cdot }{I_{rms}^{2}(t_{j})}\cdot u^{2}(\delta_{v2}^{\prime })\right]}$$

To determine the active power uncertainty calculated at the high voltage side, *P_HV_(t_j_)*, the rest of the uncertainty terms *u_2,_ u_3_ and u_4_* should be considered:

(32) $$u_{2}=\sqrt{u^{2}(SF_{VD})+\sum_{l}u^{2}(\delta_{l,VD})+\sum_{l}c_{l}^{2}\cdot u^{2}(\delta_{l,t})}$$

(33) $$u_{3}=\sqrt{u^{2}(SF_{CT})+\sum_{l}u^{2}(\delta_{{}_{l,CT}}^{})+u^{2}(R_{s})+\sum_{l}u^{2}(\delta_{{}_{l,R_{s}}}^{})+\sum_{l}c_{l}^{,2}\cdot u^{2}(\delta_{l,t}^{\prime })}$$

(34) $$u_{4}=\sqrt{\underset{l}{\sum }u^{2}\left(Sp_{l}\right)+\sum_{l}u^{2}\left(Sp_{l}^{\prime }\right)+u^{2}\left(Sp_{3}^{2}\right)}$$

##### Apparent Power

Considering Formula (10), the apparent power uncertainty can be calculated as:

(35) $$u\left[S_{HV}\left(t_{j}\right)\right]=\sqrt{{\left(\frac{\partial S_{HV}\left(t_{j}\right)}{\partial V_{RMS}}\right)}^{2}\cdot u^{2}\left[V_{RMS}\left(t_{j}\right)\right]+{\left(\frac{\partial S_{HV}\left(t_{j}\right)}{\partial I_{RMS}}\right)}^{2}\cdot u^{2}\left[I_{RMS}\left(t_{j}\right)\right]}$$

and using per unit values this formula is transformed to:

(36) $$u_{pu}[S_{HV}(t_{j})]=\sqrt{u_{pu}^{2}[V_{RMS}(t_{j})]+u_{pu}^{2}[I_{RMS}(t_{j})]}$$

##### Non-active power

Considering Formula (12), the non-active power uncertainty is:

(37) $$u[N_{HV}(t_{j})]=\frac{1}{N_{HV}(t_{j})}\cdot \sqrt{S_{HV}^{2}(t_{j})\cdot u^{2}[S_{HV}(t_{j})]+P_{HV}^{2}(t_{j})\cdot u^{2}[P_{HV}(t_{j})]}$$

and using per unit values this formula is transformed to:

(38) $$u_{pu}[N_{HV}(t_{j})]=\sqrt{{\left(\frac{S_{HV}(t_{j})}{N_{HV}(t_{j})}\right)}^{4}\cdot u_{pu}^{2}[S_{HV}(t_{j})]+{\left(\frac{P_{HV}(t_{j})}{N_{HV}(t_{j})}\right)}^{4}\cdot u_{pu}^{2}[P_{HV}(t_{j})]}$$

#### 3.2.3. Uncertainty of the Signal Processing Contributions

##### Uncertainty Contribution Due to Trapezoidal Integration Rule ($Sp_{1}$)

To achieve a null error due to numerical integration applying the trapezoidal integration rule when sinusoidal waveforms are used the Nyquist criterion must be satisfied up to and including the maximum harmonic *h_max_, n* ≥ 2 *· h_max_* + 1. For example, if *h_max_* = 100, then *n* ≥ 201 samples are required. This assumes that, for the fundamental period of 20 ms, 201 sampling intervals (202 samples) are required. Therefore, the sampling rate must be at least *f_s,min_* slightly higher than the minimum sampling rate according to the Nyquist criterion:

(39) $$f_{Nyquist}=2\cdot \frac{h_{max}}{20\;\mathrm{ms}}=2\cdot \frac{100}{20\;}=10\;\mathrm{kHz}\;\to \;f_{s,min}=\frac{202}{20\;\mathrm{ms}}=10.1\mathrm{kHz}\;$$

**Figure 9:**
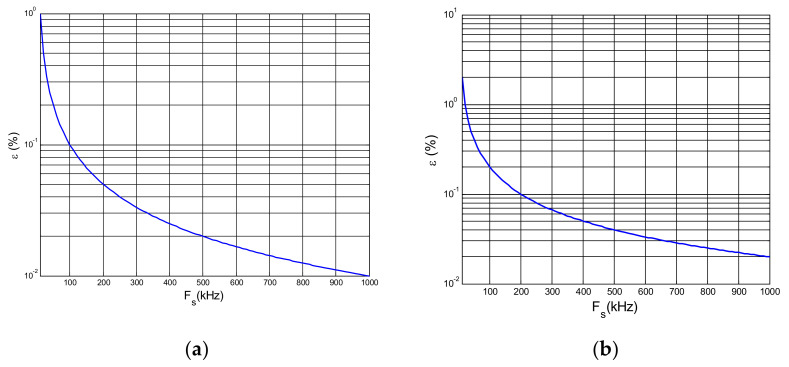
Relative error for the 90° phase-fired current waveform (50 Hz) vs. sampling rate: (**a**) of the rms and (**b**) of the active power.

However, the relative error due to the application of the trapezoidal rule when the rms value of a 90° phase-fired waveform is much bigger because of their large frequency content. This relative error can be determined as a function of the number of samples per period, *n*. For example, for a sampling rate of 500 kHz that means *n =* 10,000 when the period is 20 ms (50 Hz), the relative error is less than 0.02% (see Figure 9a).

The relative error curve for the active power measurement is twice (0.04%) when the current signal is a 90° phase-fired waveform using a sinusoidal voltage (see Figure 9b).

##### Uncertainty Contribution Due to an Integration Time Different to the Complete Period ($Sp_{2}$)

If the numeric integration applying the trapezoidal rule is performed up to an integer *n_1_* of sampling intervals different from the integer *n* of sampling intervals whose duration is the same as the entire period, a relative error is caused when rms voltage, rms current, active power, apparent power or non-active power are measured.

For sinusoidal voltage and current signals assuming *n* >> 1, the rms value relative error is given by the following analytical formula:

(40) $$\varepsilon \approx (\lambda -1)-\frac{cos2\cdot \left(2\cdot \pi \cdot f\cdot t_{j}+\lambda \cdot \pi \right)\cdot sin(2\cdot \pi \cdot \lambda )}{2\cdot \pi }$$

where *λ* = *n*_1_/*n*.

**Figure 10:**
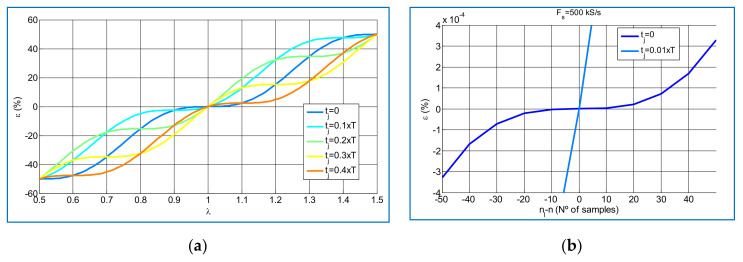
Relative error *ε* of the rms value of a digitized sinusoidal waveform due to integrating in an integer n_1_ of sampling intervals different from the integer n of sampling intervals whose duration is the same as the entire period: (**a**) *ε(%)* vs. *λ*; (**b**) *ε(%)* vs. *n_1_* − *n*.

The curve family of the relative error, *ε*, expressed in%. for different starting times *t_j_* is shown in Figure 10a. The starting time *t_j_* should be close to zero, while *λ* should be as close as possible to 1 as much as possible to achieve a negligible error. For instance, for *t_j_ =* 0.01·T and *n =* 10,000 (*f_s_ =* 500 kHz for *f =* 50 Hz) the relative error is less than 5 × 10^−5^ % *(ε <* 5 × 10^−5^ %). When the difference between *n_1_* and *n* is known and *t_j_* ≤ 0.01·T, the relative error can be determined using the curve shown in Figure 10b. For example, if *n_1_* − *n =* 10 samples (*λ* = 0.999 for Figure 10a) then *ε* < 5 × 10^−5^ %.

The following analytical formula for the relative error of the measured active power of sinusoidal voltage and current signals with a *ϕ* phase shift can be determined, assuming *n* >> 1:

(41) $$\varepsilon =\left(\lambda -1\right)-\frac{cos2\cdot \left[(2\cdot \pi \cdot f\cdot t_{j}+\lambda \cdot \pi )-\varphi \right]\cdot sin(2\cdot \pi \cdot \lambda )}{2\cdot \pi }$$

For *ϕ = 0* this formula is transformed to (39) and the same conclusions as the ones achieved for rms voltage and current measurements can be extended for the active power.

In the case of a 90° phase-fired waveform current signal using a sinusoidal voltage, same relative error *ε <* 5 × 10^−5^ % is obtained by numerical methods, for *n_1_* − *n =* 10 samples.

##### Uncertainty Due to the Synchronization Error between Multimeters ($Sp_{3}$)

**Figure 11:**
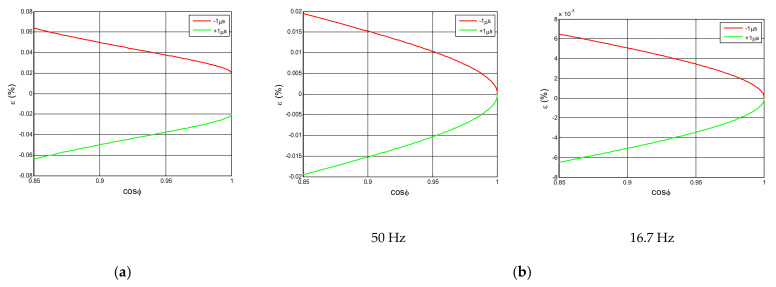
The relative error of the calculated active power expressed in %, due to the synchronization multimeters error ±1 μs: (**a**) Sinusoidal (50 Hz) voltage and 90° phase-fired current waveform; (**b**) Sinusoidal current with low harmonic content (THD < 2%) with sinusoidal voltage with 10% for *h* = 5th and 3% for *h* = 11th.

Considering that the maximum synchronization error between the digital multimeters is ±1 μs, the relative error of the active power expressed in (%) due to this synchronization error of ±1 μs for the *cosφ* range between from 0.85 and 1 is calculated. Figure 11a shows the error trend for 90° phase-fired current waveform and Figure 11b for sinusoidal current waveform with a low harmonic content (THD < 2%) and in both cases for a sinusoidal voltage signal with harmonic content of 10% for the 5th harmonic and 3% for the 11th harmonic.

## 4. Application of the Uncertainty Estimation of the EMS Calibration Set Up

Applying Formulas (19) and (20), the rms voltage and current uncertainties can be determined. Based on these results, the apparent power uncertainty can be determined by applying Formula (36). The active power uncertainty is given by Formula (24) and the non-active power uncertainty is calculated by applying Formula (38). Table 2 shows a summary of all calculated expanded uncertainties for the EMS calibration set up developed by LCOE, for 90° phase-fired current waveform. For this waveform, a sensitivity coefficient of −6.26 × 10^−5^%/μrad was obtained from the relative error of −0.01966% obtained for 1 μs shift between voltage and current signals of a 90° phase-fired current waveform, corresponding to *cosφ* = 1, as shown in Figure 11a. Therefore, taking into account that 1 μs corresponds to 314.16 μrad for 50 Hz signal, the sensitivity coefficient is equal to −0.01966%/314.16 = −6.26 × 10^−5^%/μrad.

The uncertainty estimation of the EMS calibration set up developed by LCOE is analyzed in detail for active power measurements by applying Formulas (31)–(34) (see Table 3). The uncertainty contributions due to independent low voltage sampling of both voltage and current signals are given by Formula (31), in which the sensitivity coefficients corresponding to the constant errors for voltage and current measurements, *δ_v1_* and *δ’_v1_*, are 1 and the ones for the digitizer variable errors, *δ_v2_* and *δ’_v2_*, depend on the voltage or current rms value to be measured by the DMMs, which are assumed to be higher than the 10% of the full scale in both cases. The uncertainty contributions due to the relation between the high voltage and current scale factors are given by Formulas (32) and (33), respectively. The standard uncertainties of the different influence parameters such as drift, temperature coefficient, short term stability, non-linearity and frequency dependence, for voltage divider, current transducer and shunt, are collected from the manufacturer data sheet. A rectangular probability distribution is assumed in this case. Influence parameter such as the voltage divider, current transducer and shunt calibration uncertainties are collected from the calibration certificates, assuming a normal probably distribution. The standard uncertainties related to signal processing method are obtained by applying Formula (34) from the analysis carried out in Section 3.2.2. The standard uncertainty corresponding to the trapezoidal integration method, *Sp_1_* and *Sp’_1_*, depends on the sampling rate. For example, assuming a rectangular probability distribution, for *f_s_ =* 500 kHz it is *0.04%* for active power (Figure 9). This total error contribution is split in two equal contributions for both the voltage and the current, equal to √2 × 0.04%/2 = 0.028% (*Sp_1_ =* 0.028% and *Sp_2_* = 0.028%). The standard uncertainty due to integrating in an integer n_1_ of sampling intervals different from the integer n of sampling intervals whose duration is the same as the entire period, *Sp_2_* and *Sp’_2_*, is determined by applying Formula (40). Assuming a rectangular distribution, if the difference between the acquired number of samples and the number of samples corresponding to a complete period is 10 samples, when *n =* 10,000 samples and the sampling rate, *f_s_*, is 500 kSamples/s, the relative error is less than 5 × 10^−5^. Finally, the standard uncertainty due to the synchronization between both digital multimeters, *Sp_3_*, is derived of the manufacturer data sheet. Considering the maximum synchronization time given by the manufacturer of 1 μs, and assuming a rectangular probability distribution, this standard uncertainty results in a value of 314μrad/√3. From the uncertainty contributions referred in Table 3 and applying Formula (24), the active power expanded uncertainty is lower than 0.3%.

## 5. Conclusions

A new EMS calibration set up is available for AC voltages up to 25 kV addressed to on board energy metering under distorted conditions. It consists of a phantom power generator composed of two independent synchronized sources capable of generating high voltages with superimposed harmonics components up to 5 kHz, and a phase-fired current waveform or a sinusoidal current up to 500 A with harmonics up to 5 kHz, according to the EN 50463-2 requirements.

Based on two synchronized digital multimeters, to sample the voltage and current waveforms to be measured, two precise traceable measuring systems, one for high voltage up to 15 kV (16.7 Hz) or 25 kV (50 Hz) and the other for high current up to 500 A, with a bandwidth up to 5 kHz, were developed.

Function models were introduced for rms voltage, rms current and power quantities (active, apparent and non-active) and analytical formulas have been derived to analyze the uncertainty contribution due to different influence parameters. Particular attention was paid to uncertainty contributions of signal processing, such as integration by applying the trapezoidal rule, the integral at a set of number of sampling intervals different to the full period duration, and synchronization error between digital multimeters. This uncertainty analysis provides an expanded uncertainty for the EMS calibrations for active power measurement and apparent power better than 0.3%, and for non-active power better than 0.5% according to the target of the “MyRailS” European project [5,6,7,8], solving the traceability lack for this type of measurements.

## Figures and Tables

**Table 1:** Reference measuring systems.

Measuring System	Component	Type	Performance
Voltage	Resistive divider	LCOE	1060/1
	Measuring cable	RG-58	50 Ω, 4 m
	Matching impedance	LCOE	1.1 MΩ
	Multimeter (Master)	Keithley DMM7510	700 V, 10 A, 1 MS/s
Current	Fluxgate current sensor	LEM ITN-900-S	636 A, 1500/1 A
	DC source	LCOE	±15 VDC
	Shunt	LCOE	10 Ω
	Multimeter (Slave)	Keithley DMM7510	700 V, 10 A, 1 MS/s
Energy calculation	Software	LCOE	

**Table 2:** Summary of expanded uncertainties *U* (*k* = 2).

Expanded Uncertainty	%
U[P] (%)	0.23
U[V_RMS_] (%)	0.15
U[I_RMS_] (%)	0.10
U[S] (%)	0.18
U[N] (%)	0.43

**Table 3:** Uncertainty budget for the active power for 90° phase-fired current waveform.

Influence Parameters *X_i_*	Symbol	Estimate	Standard Uncertainty *u(x_i_)*	Probability Distribution	Sensitivity Coefficient *c_i_*	Uncertainty Contribution *u_i_(y)*
Digitization of Voltage If *V_RMS_(t_j_)* = 10% *V_FS_*	$\delta_{v1}\%$	0	0.012/√3% ^(I)^	Rectangular	1	0.0069%
	$\delta_{v2}\%$	0	0.0075/√3% ^(I)^	Rectangular	$\sqrt{0.82}\cdot 10=9.06$	0.039%
Digitization of Current If *I_RMS_(t_j_)* = 10% *I_FS_*	$\delta \prime_{v1}\%$	0	0.012/√3% ^(I)^	Rectangular	1	0.0069%
	$\delta \prime_{v2}\%$	0	0.0075/√3% ^(I)^	Rectangular	$\sqrt{1.62}\cdot 10=12.73$	0.055%
VD SF calibrat. uncertainty	$SF_{VD}$	1059	0.1/2% ^(II)^	Normal	1	0.05%
VD SF drift	$\delta_{1\mathrm{VD}}$	0	0.02/√3% ^(I)^	Rectangular	1	0.011%
VD temperature coefficient	$\delta_{2,\mathrm{VD}}$	0	0.0075/√3% ^(I)^	Rectangular	1	0.0043%
VD short term stability	$\delta_{3,\mathrm{VD}}$	0	0.06/√3% ^(I)^	Rectangular	1	0.035%
VD non-linearity	$\delta_{4,\mathrm{VD}}$	0	0.03/√3% ^(I)^	Rectangular	1	0.017%
VD phase uncertainty	$\delta_{1,t}$	0	58/2 μrad ^(II)^	Normal	−6.26 × 10^−5^%/μrad	0.0018%
VD residual phase error	$\delta_{2,t}$	0	1452/2 μrad ^(II)^	Normal	−6.26 × 10^−5^%/μrad	0.045%
CT SF calibration uncertainty	$SF_{CT}$	1500	0.0070/2% ^(II)^	Normal	1	0.0035%
CT SF drift	$\delta_{1,CT}$	0	0.005/√3% ^(I)^	Rectangular	1	0.0029%
CT temperature coefficient	$\delta_{2,CT}$	0	0.006/√3% ^(I)^	Rectangular	1	0.0034%
CT SF non-linearity	$\delta_{3,CT}$	0	0.0036/√3% ^(I)^	Rectangular	1	0.0021%
CT S. F. phase calibration	$\delta \prime_{3,t}$	0	58/2 μrad ^(II)^	Normal	−6.26 × 10^−5^%/μrad	0.0018%
CT S. F. phase error drift	$\delta \prime_{4,t}$	0	293/√3 μrad ^(I)^	Rectangular	−6.26 × 10^−5^%/μrad	0.0106%
CT residual phase error	$\delta \prime_{5t}$	0	668/2 μrad ^(II)^	Normal	−6.26 × 10^−5^%/μrad	0.021%
Shunt calibration uncertainty	$R_{s}$	9.979 Ω	0.05/2% ^(II)^	Normal	1	0.025%
Shunt drift	$\delta_{1,R}$	0	0.01/√3% ^(I)^	Rectangular	1	0.0057%
Shunt temperature coeffic.	$\delta_{2,R}$	0	0.05/√3% ^(I)^	Rectangular	1	0.029%
Shunt resistance variation vs. frequency	$\delta_{3,R}$	0	0.01/√3% ^(I)^	Rectangular	1	0.0057%
Shunt residual phase error	$\delta \prime_{6,t}$	0	Negligible	Normal	1	0%
V integration trapezoidal rule	$Sp_{1,t}$	0	0.028/√3% ^(III)^	Rectangular	1	0.0161%
I integration trapezoidal rule	$Sp\prime_{1,t}$	0	0.028/√3% ^(III)^	Rectangular	1	0.0161%
V Samples taken for a period	$Sp_{2,t}$	0	5·10^−5^/√3% ^(IV)^	Rectangular	1	0.00003%
I Samples taken for a period	$Sp\prime_{2,t}$	0	5·10^−5^/√3% ^(IV)^	Rectangular	1	0.00003%
Synchroniz. between DMM	$Sp_{3,t}$	0	314μrad/√3 ^(V)^	Rectangular	−6.26 × 10^−5^%/μrad	0.011%
Combined variance	$u_{c}^{2}(y)=\sum u_{i}^{2}(y)$					0.0136%
Combined standard uncert.	$u_{c}^{}(y)$					0.117%
Expanded uncertainty	$U[P](\%)=\kappa \cdot u_{c}^{}(y)(\kappa =2)$					0.23%

(I) Data from manufacturer data sheet. (II) Data from calibration certificate. (III) As indicated in Uncertainty Contribution Due to Trapezoidal Integration Rule ($Sp_{1}$), an error of 0.04% for active power is obtained for 90° phase-fired waveform using a sinusoidal voltage. This total error contribution is split in two equal contributions for both the voltage and the current, equal to √2 × 0.04%/2 = 0.028%. (IV) The value of 5 × 10^−5^% comes from Uncertainty Contribution Due to an Integration Time Different to the Complete Period ($Sp_{2}$). (V) 314μrad corresponds to the maximum synchronization error between the digital multimeters of 1 μs for 50 Hz, as indicated in Uncertainty Due to the Synchronization Error between Multimeters ($Sp_{3}$).
